# Juxta-Vesical Urinary Stones: An Extremely Rare Finding Secondary to Bladder Rupture and Squamous Cell Carcinoma in a Patient on Clean Intermittent Self-Catheterization

**DOI:** 10.7759/cureus.38776

**Published:** 2023-05-09

**Authors:** Ioannis Tsikopoulos, Dimitrios Papadopoulos, Asterios Symeonidis, Stamatios Katsimperis, Chrysovalantis Gkekas

**Affiliations:** 1 Department of Urology, General University Hospital of Larissa, Larissa, GRC; 2 Department of Urology, West Middlesex University Hospital, Isleworth, GBR; 3 Department of Medicine and Surgery, Aristotle University of Thessaloniki, Thessaloniki, GRC; 4 Department of Urology, National and Kapodistrian University of Athens, Sismanogleio General Hospital, Athens, GRC; 5 Department of Urology, 404 Military Hospital, Larissa, GRC

**Keywords:** bladder rupture, intermittent catheter, urology and oncology, squamous cell carcinoma (scc), urinary lithiasis

## Abstract

We present a rare case of juxta-vesical urinary stones in the lesser pelvis, incidentally diagnosed during the investigation of a urinary tract infection (UTI). The patient (male) had a history of neurogenic bladder and performed self-catheterizations. After the initial workup, the patient was admitted with a complicated UTI diagnosis. CT scan of the abdomen and pelvis depicted multiple bladder stones, some calculi lying juxta- and retro-vesically, an abscess cavity, and diffuse thickening of the bladder. The abscess was adherent to the bladder wall, containing calculi, too. We presumed that the patient self-inflicted a bladder rupture when performing clean intermittent self-catheterization (CISC) and stones dislodged in the pelvis due to his poor bladder sensation. Flexible cystoscopy was attempted but was not completed due to stone obstruction and poor bladder compliance. The patient underwent open surgical exploration. Several calculi were removed, the abscess was drained, and bladder wall biopsies were taken. Pathology results revealed invasive squamous bladder carcinoma; the patient was listed for radical cystectomy. We aim to familiarize the clinician with rare complications that should be taken into consideration when treating patients on CISC and present an extremely rare clinical finding of juxta-vesical lithiasis.

## Introduction

Juxta-vesical urolithiasis is extremely uncommon, and it represents the dislodgement of a urinary calculus that could either be traumatic or iatrogenic. To our knowledge, this is the first case report describing such morbidity. Bladder lithiasis, on the other hand, is rare as it accounts for almost 5% of all urinary tract stones. Either it is multifactorial and linked to urinary bladder pathologies (the most common being bladder outlet obstruction), or it represents a passage of an upper urinary tract calculus. Approximately 21% to 78% of bladder stones can be seen on a plain kidney, ureter, and bladder (KUB) X-ray, and this is usually the first investigation used [1]. Oftentimes, bladder stones can be seen more laterally than usual on an X-ray film, which is common for stones nested within a bladder diverticulum, but still, they have to be differentiated from extravesical mimics such as phleboliths, ingested foreign bodies, calcified metastatic implants, migrated gallstones, or peritoneal loose bodies [2].

## Case presentation

A 50-year-old male patient presented to the emergency department (ED) with fever, rigors, and lower urinary tract symptoms (LUTS) since the previous day. He did not complain of nausea or diarrhea. The patient mentioned a medical history of clean intermittent self-catheterization (CISC) for the past 15 years due to either anatomical or functional obstruction but was not been investigated to that day with urodynamic studies. His temperature was 38.7 °C, and he was slightly tachycardic (BP 145/87, heart rate [HR] 101 beats per minute, SpO2 98%, and respiratory rate [RR] 28 breaths per minute). According to his qSOFA score (1 point), he was at low risk but presented two of the total four Systematic Inflammatory Response Syndrome (SIRS) criteria. Physical examination was positive only for mild suprapubic discomfort upon palpation, and he had no flank pain. The patient was admitted with the diagnosis of a complicated urinary tract infection (UTI; a complication of bladder lithiasis). In his initial investigation, C-reactive protein (CRP) was 35 mg/L (reference range 0-5 mg/L), his full blood count (FBC) demonstrated leukocytosis (WBC 14,200), and his urine tested positive for blood, leucocytes, and nitrates, indicating UTI. Blood and urine cultures were also collected (urine culture positive for *Escherichia coli*). He was initially started on empirical intravenous antibiotics and adjunctive measures for sepsis. Later, on microbiology advice, definitive antibiotics were intravenously administered (*E. coli* sensitive to quinolones). His blood parameters were monitored daily. On the fifth day of admission, he became afebrile and his clinical condition was markedly improved. The laboratory findings are summarized in Table 1.

**Table 1:** Laboratory workup. WBC, white blood cells; CRP, C-reactive protein

Lab	Value	Reference range
WBC count	14.2	4,000-11,000 μL^-1^
Absolute neutrophils	9.25	2,000-7,500 μL^-1^
Hemoglobin	10.4	13.5-17 g/dL
Hematocrit	30.7	40%-52%
Platelet count	330	150,000-400,000 μL^-1^
Serum creatinine	1.3	0.8-1.3 mg/dL
Serum urea	60	10-50 mg/dL
CRP	35	0-5 mg/L
Serum potassium	3.7	3.5-5 mEq/L
Serum sodium	137	136-146 mEq/L

**Figure 1:**
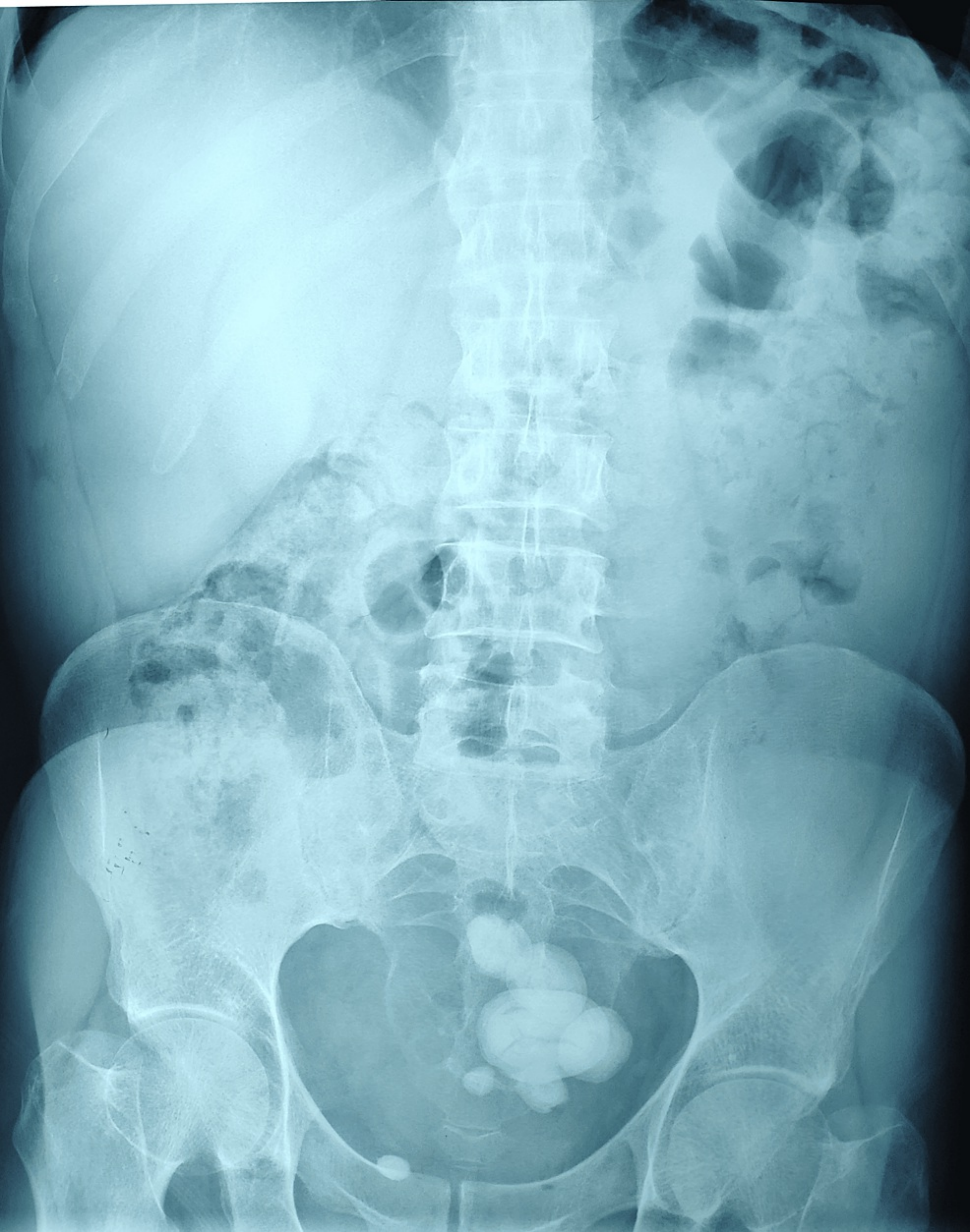
Plain film of the abdomen showing giant vesical calculi.

Ultrasound and KUB X-ray, performed in the ED, revealed multiple stones in the bladder, hyperplastic prostate (50 cc), and no hydronephrosis (Figure 1). Αn indwelling 16Fr Foley catheter (Teleflex®, Kamunting, Malaysia) was inserted, and 350 mL of turbid urine was emptied. Observing the KUB X-ray, one could easily notice that some of the stones were very lateral, which could implicate a bladder diverticulum and necessitate further investigation.

**Figure 2:**
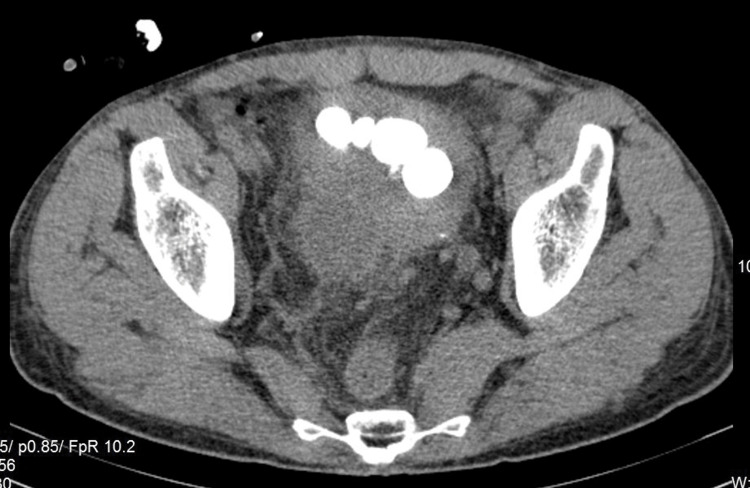
Axial plane of contrast CT scan showing multiple vesical calculi. CT, computed tomography

**Figure 3:**
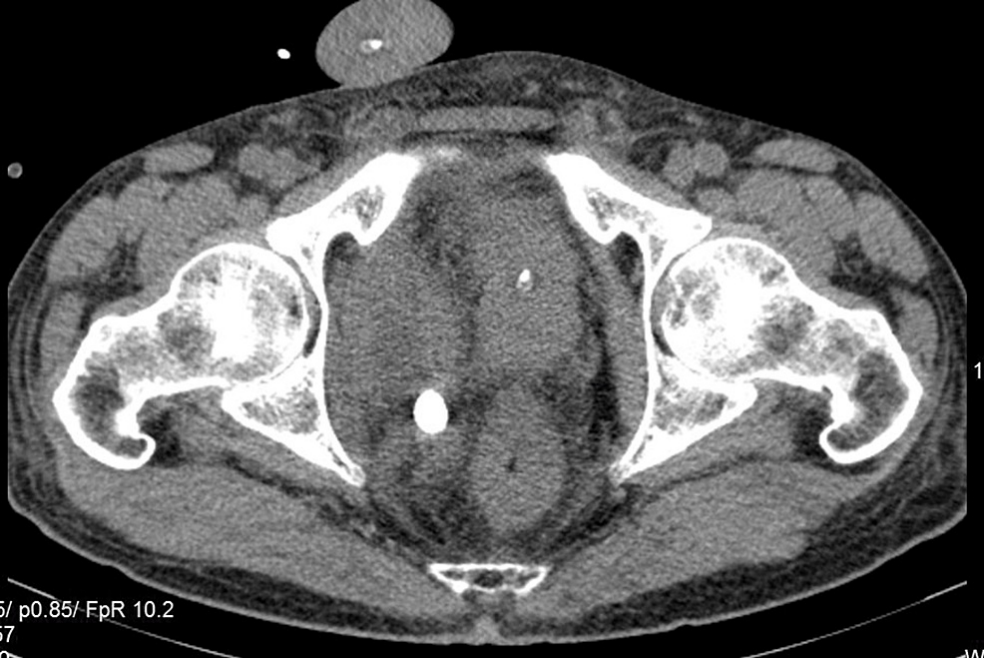
Axial plane of contrast CT scan depicting extravesical calculus. CT, computed tomography

**Figure 4:**
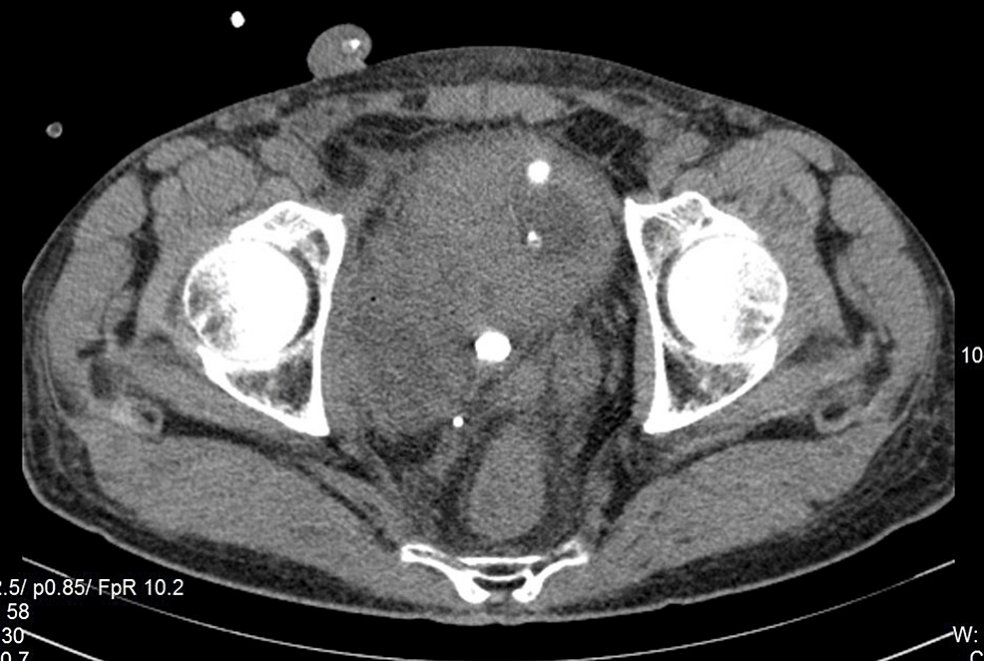
Axial plane of the contrast CT scan showing both extravesical and vesicle calculi. CT, computed tomography

The CT scan of the abdomen and pelvis was subsequently performed, which demonstrated multiple bladder stones (round shaped with an average of 890 Hounsfield Units), a few round stones of similar density lying juxta- and retro-vesically complicated with local inflammation, abscess formation near the posterior bladder wall containing calculus, and presumably pus and diffuse thickening of the bladder, more prominent on the anterior wall (Figures 2-4). The patient was scheduled for cystolitholapaxy 30 days postdischarge.

A month later, the patient presented for his scheduled surgery. During the procedure, it was difficult to inspect the bladder due to the presence of multiple sizeable calculi that interfered with the scope and inadequate bladder filling, which gave the impression of compromised compliance, not unusual in neurogenic bladders. Flexible cystoscopy was thus not completed due to the stone obstruction and reduced bladder compliance. But even more stressful was the impression we had that not every stone lay intravesically. Therefore, an intraoperative cystography under fluoroscopy was performed, to detect a possible diverticulum or a rupture. The cystography failed to detect a leakage of the contrast or a sacculation of the bladder. It, however, confirmed the reduced compliance and the small bladder capacity but, even worse, demonstrated opacities beyond the bladder contour.

Open surgical exploration was performed for the removal of the stones and the drainage of the abscesses. Pus with stones was evacuated from the lesser pelvis and sent for culture. Biopsies from the thickened bladder wall areas were also taken intraoperatively. They revealed squamous cell carcinoma (SCC), and subsequently, the patient was scheduled for radical cystectomy, a month later.

A multidisciplinary team (MDT) discussion followed, and the patient opted for radical cystectomy (an ileal conduit), which was performed after one month uneventfully. Unfortunately, two months after the radical cystectomy, we were informed that the patient was hospitalized in a local care unit for sepsis due to a multi-drug-resistant bacteria infection. He died of sepsis during that admission.

## Discussion

This is the first reported case of incidental finding of juxta-vesical calculi, to our knowledge, and highlights the need for proper follow-up of urologic patients. A patient with no urologic follow-up presented to the ER for fever, but the underlying problem was much more serious. The causes that lead to the formation of the calculus-containing abscess are unclear. It could be due to ill-performed self-catheterizations that lead to a traumatic rupture of the bladder. This, in turn, leads to the dislodgement of a few stones and an accompanying infected urinoma, which runs unnoticed owing to the reduced sensitivity of the bladder (secondary to an undiagnosed neuropathy). This neurological condition is, on the other hand, the reason for the initial incorrect execution of the self-catheterization due to the absence of tactile feedback from the bladder.

Al Edwan et al. reported a case of a patient with SCC of the bladder who presented with spontaneous intraperitoneal bladder rupture [3]. Other causes of spontaneous bladder rupture are reported by Sawalmeh et al., including inflammation, alcoholism, radiotherapy, and neurogenic bladder [4]. In every case, there is an underlying pathology that weakens the bladder wall and precipitates perforation. In our case, the large vesical calculi along with the self-catheterizations and the underlying malignancy led to increased pressure against the noncompliant bladder and a possible perforation. This is only a hypothesis as our clinical findings were not indicative of a recent rupture (absence of contrast leakage on cystography), but the abscess was adherent to the posterior bladder wall on the CT scan [5].

Invasive SCC has been linked to chronic inflammation and UTIs. A direct association between bladder cancer (BC) and UTIs has been addressed in several case-control studies, which reported a twofold increased risk of BC in patients with recurrent UTIs [6]. This contradicts a recent meta-analysis that showed no statistical association between UTIs and SCC BC and highlights the need for further evidence regarding the potential correlation [7]. However, there is an established correlation between self-catheterizations and SCC [8]. Other possible risk factors include urinary calculi and chronic inflammation of the urothelium [9]. In our case, both vesical calculi and intermittent self-catheterizations (ISCs) and possibly chronic inflammation of the bladder were present. One has every reason to believe that this unpleasant occurrence could have been avoided through routine screening of catheterized patients with regular urinary cytology, cystoscopy combined as needed with biopsies (random or targeted), and ultrasound examination [10]. Further studies on the subject are needed to conceptualize such a screening.

The strengths of our approach include the quick medical adaptations and changes in surgical plans due to complications and the uniqueness of this case. There are also some limitations regarding our case, as our patient had an unclear history of neurogenic bladder, not confirmed by any urodynamic studies and he did not show up for his follow-ups with his urologist for many years. Also, we had no cystoscopy results and images due to reduced compliance of a bladder filled with calculi, which obstructed the procedure. We believe that many of the complications and the presented comorbidities could be avoided with proper previous follow-up and monitoring.

## Conclusions

ISCs may be connected to malignant histopathological changes in the bladder like SCC. Neglected bladder calculi, neurogenic bladder, chronic inflammation, and even SCC may precipitate the weakening of the bladder wall and ultimately lead to the rupture of the bladder. Perforation of the bladder in patients with neurogenic bladder on ISCs can be asymptomatic and eventually lead to the formation of an abscess cavity. Finally, we advocate the use of routine screening for neurogenic bladder patients performing ISCs.
